# Letter to the editor on: “Meta-analysis of the histopathological features of surgically removed aortas classified according to the SCVP and AECVP consensus documents”

**DOI:** 10.1016/j.ahjo.2026.100970

**Published:** 2026-10-16

**Authors:** Gursimar Pal Singh, Bhumika Vaishnav

**Affiliations:** a Dr. D. Y. Patil Medical College, Hospital and Research Centre, Pimpri, Pune, India; b Department of Medicine, Dr. D. Y. Patil Medical College, Hospital and Research Centre, Pimpri, Pune, India

**Keywords:** Aortic pathology, Aortic aneurysm, Aortic dissection, Bicuspid aortic valve, Medial degeneration, Histopathology, SCVP consensus, AECVP consensus, Prevalence meta-analysis, Meta-regression

Dear Editor,

Jämsén et al. [1], pool histopathological gradings of 1282 thoracic aortic specimens assessed against the SCVP and AECVP criteria and conclude that dissected aortas carry more severe medial degeneration than aneurysmatic ones, with bicuspid valve aortas mildest of all. Mapping how the consensus scheme is used in practice is worth doing. However, the pooled estimates as published will not carry out that comparison.

Several reported confidence intervals fall outside the range of prevalence. Elastic fiber disorganization in the aneurysm cohort was 47.5% with an interval of −47.5% to 135.6%, and in the whole cohort, it was 30.5% with a lower bound of −0.3% [2]. Translamellar medial fibrosis reached 102.2% in dissections and 117.5% in bicuspid aortas, and laminar medial collapse reached 100.8%. This follows from applying the DerSimonian–Laird method to the untransformed proportions [3]. Pooling on the logit scale, or after a Freeman–Tukey double-arcsine transformation, keeps estimates inside their natural bounds and is the usual approach for prevalence meta-analyses.

One interval is incompatible with its own estimate. Moderate medial degeneration in the dissection cohort was reported as 47.1% with a 95% interval of 5.7% to 30.6%. The same interval was given for mild degeneration in the aneurysm cohort; therefore, it appears to have been carried over from a different analysis and it is the principal finding of the study.

The grade distributions also failed to reconcile. Mild degeneration in the aneurysm cohort appears as 8.2% in the Results and as 18.2% in the summary of severities. Severe degeneration in dissections is 37.2% in two places and 34.5% in a third. The four bicuspid grades sum to 104.5%, and absent degeneration there is reported as 0.8% although no such case was observed, which presents the 0.5 continuity correction as a result. Atherosclerosis is described as present in 52.5% of 670 samples, while 52.5% of 640 samples is the value given for its absence. Laminar medial collapse is present in 46.5% and absent in 57.5%. Denominators move as well: the dissection cohort is defined as 422 samples from six studies, yet the degeneration analysis rests on 264 from five, and the bicuspid cohort of 284 becomes 78, then 76, then 75.

No formal comparison between cohorts was performed. The severity ordering was read off separately pooled subgroups with overlapping intervals, several carrying inconsistency indices above 95% and some approaching 99.9%, whose specimens are not independent because single reports feed two or three cohorts at once. Ranking the cohorts requires a subgroup interaction test or meta-regression [4].

The eligibility rule compounds this. Reports were set aside for grading on a 0–4 scale rather than by frequency, among them series of 496, 400, 217, and 141 specimens; thus, nearly as many aortas were discarded over reporting format as were analyzed. Ordinal gradings can be pooled as standardized mean differences instead of dropped.

The question of whether the consensus components distinguish these conditions deserves further investigation. Answering it requires the analysis to be rerun on a bounded scale with these discrepancies resolved.

## CRediT authorship contribution statement

**Gursimar Pal Singh:** Writing – review & editing, Writing – original draft, Visualization. **Bhumika Vaishnav:** Conceptualization, Data curation, Funding acquisition.

## Informed consent

Not applicable.

## Ethical approval

Not applicable (Commentary on a previously published article).

## Declaration of Generative AI and AI-assisted technologies in the writing process

Generative AI tools were used only for language refinement and formatting assistance. All scientific interpretation, critical appraisal, and manuscript preparation were performed independently by the authors.

## Funding

None.

## Declaration of competing interest

The authors declare that there are no conflicts of interest.

## Data Availability

No new data were generated or analyzed in this study.
